# Accurate Anisotropic Fast Marching for Diffusion-Based Geodesic Tractography

**DOI:** 10.1155/2008/320195

**Published:** 2007-12-06

**Authors:** S. Jbabdi, P. Bellec, R. Toro, J. Daunizeau, M. Pélégrini-Issac, H. Benali

**Affiliations:** ^1^Laboratoire d'Imagerie Fonctionnelle, INSERM, U678, 75013 Paris, France; ^2^Faculté de Médecine Pitié Salpêtrière, Université Pierre et Marie Curie, UMR 678 CNRS, 75013 Paris, France; ^3^Oxford Centre for Functional Magnetic Resonance Imaging of the Brain (FMRIB), Oxford OX3 9DU, UK; ^4^McConnell Bain Imaging Center, Montreal Neurological Institute, McGill University, Montréal, Canada H3A 2T5; ^5^Brain & Body Centre, The University of Nottingham, Nottingham NG7 2RD, UK; ^6^Functional Imaging Laboratory, University College London, London WC1E 6BT, UK; ^7^Unité d'Imagerie Fonctionnelle, Université de Montréal, Montréal, Canada H3C 3J7

## Abstract

Using geodesics for inferring white matter fibre tracts from diffusion-weighted MR data is an attractive method for at least two reasons: (i) the method optimises a global criterion, and hence is less sensitive to local perturbations such as noise or partial volume effects, and (ii) the method is fast, allowing to infer on a large number of connexions in a reasonable computational time. Here, we propose an improved fast marching algorithm to infer on geodesic paths. Specifically, this procedure is designed to achieve accurate front propagation in an anisotropic elliptic medium, such as DTI data. We evaluate the numerical performance of this approach on simulated datasets, as well as its robustness to local perturbation induced by fiber crossing. On real data, we demonstrate the feasibility of extracting geodesics to connect an extended set of brain regions.

## 1. INTRODUCTION

For decades, dissection, lesion studies, or axonal transport of tracers have been the only available techniques for studying the brain's anatomical connections. It is not surprising that due to the invasiveness of these methods, most of the data concerning the large-scale, white matter tracts of the brain were collected on animals, for example, cats [1] or monkeys [2], while structural data for the human brain were largely missing [3]. Diffusion weighted MR imaging now offers a propitious and unique framework to explore noninvasively the organisation of white matter in the living human brain [4, 5]. Despite the poor spatial resolution of this technique, already diffusion data are beginning to inform us about human brain large-scale connections [6–8] and how they relate to the functional role of cortical and subcortical networks [9, 10].

Inferring on white matter architecture from diffusion data relies on the properties of water diffusion in the tissues. Water molecules diffuse more easily along the fibre tracts than across them, and this anisotropy is captured by the diffusion-weighted MR signal. Inferring on connexions given this local feature is challenging, since the observations (diffusion properties) are indirectly related to the actual structure (axonal orientations, size, and packing). The tractography algorithms use the information of directionality contained in diffusion data to infer connectivity between brain regions. Usually, information about the orientation of white matter fibres is estimated locally, via models (e.g., diffusion tensor imaging (DTI) [11], mixture models [12], or partial volume models [13, 14]) or in a model-free manner (e.g., Q-ball imaging [15]). Fibre tracking consists then in inferring connexions between distant brain regions, given this local orientation. This can be done either in a deterministic way, by trusting the local orientation information and following these directions until reaching a target region (i.e., streamline tractography [16–19]), or in a probabilistic way, by building distributions of connexions, using local probabilistic models for fibre orientation distributions [13, 14, 20].

In both cases, when tracking a fibre between two regions of the brain, these algorithms start in one seed region, and try to find the tracts, or distribution of tracts, that will end up in the target region. In cases where the local orientation information present in the diffusion data is consistent with the presence of this pathway, then these tractography algorithms manage in general to recover the connexion between the seed and the target. However, it often happens that in some parts of the trajectory, the local diffusion information no longer supports the presence of the pathway. This can either be due to a high level of noise compared to the actual signal, or to the presence of a high number of crossing fibres heterogeneous in their orientations. This issue is crucial in streamlining algorithms, and is also met in probabilistic algorithms when a single orientation per voxel is modelled [21]. The problem with those algorithms is that when tracking from a seed, the algorithm has no information about the region it will end up in.

A possible solution to the problem of local perturbations in the diffusion data may be provided by global tractography, that is, optimising a global criterion while seeking for connexions. A global tractography algorithm can potentially overcome errors in estimating local structure, because its goal is to connect two given regions. In other words, if we tell the algorithm which connexion we are looking at, that is, which pair of regions is to be connected, it is better at finding it. Geodesic tractography (GT), first proposed by Parker et al. [22], falls into this category. GT is based on the hypothesis that brain fibers can be interpreted as minimal distance paths (geodesics) for a metric derived from the water diffusion profile. This distance criterion is global by definition.

The basic idea for constructing a geodesic in a metric space is to build a distance field from a seed region, the very same region one would use as a seed for streamline tractography. This is done by solving the so-called Eikonal equation, a partial differential equation (PDE) that describes the time of arrival at each point of the space, as a function of the local speed. In a constant speed field, this PDE can be easily integrated, and the geodesics are simply straight lines. When the speed varies across the space, the geodesics can curve, preferring high local speed locations to decrease the arrival time. Finally, if the speed depends on the direction of travel (e.g., along versus across a fibre tract), then the PDE is said to be anisotropic.

Solving the Eikonal equation in a heterogeneous and highly anisotropic medium, as is the human brain, is a technically challenging problem [23]. This is especially true if one uses single-pass algorithms, which is particularly important when dealing with data containing hundreds of thousands of voxels. There have been a few attempts at solving this problem in the context of diffusion-based tractography [22, 24–27].

We describe a method for constructing geodesics in an anisotropic medium, and apply it to the problem of DTI-based tractography. This method relies on works in optimal path planning [28] and, more recently, vessel extraction in 3D angiography images [29]. It has been shown to be very accurate in anisotropic media [29], and requires less computation than the exact method proposed in Sethian and Vladimirsky [30] in a general framework for anisotropic optimal path planning. The main contribution of this work is to show how this method applies to the case of an elliptic medium, where the algorithm performs extremely well both in terms of accuracy and efficiency, as shown in the simulations. We also show the feasibility of applying such method to the extraction of structural connectivity in an extended brain network using diffusion data from a healthy subject.

## 2. METHODS

In this section, we will give some theoretical background on geodesics and the Eikonal equation, and describe a single-pass algorithm for building geodesics.

### 2.1. Geodesics and the Eikonal equation

A geodesic is a pathway minimising an integral of the form

(1) $$J(\gamma )=\int F(s,\gamma ,\gamma^{\prime })\text{d}s,$$

where $F(s,\gamma ,\gamma^{\prime })=\sqrt{\gamma^{\prime }({s)}^{T}M(\gamma (s))\gamma^{\prime }(s)}$ describes an infinitesimal distance along a pathway γ, relative to a metric tensor M.

**Figure 1:**
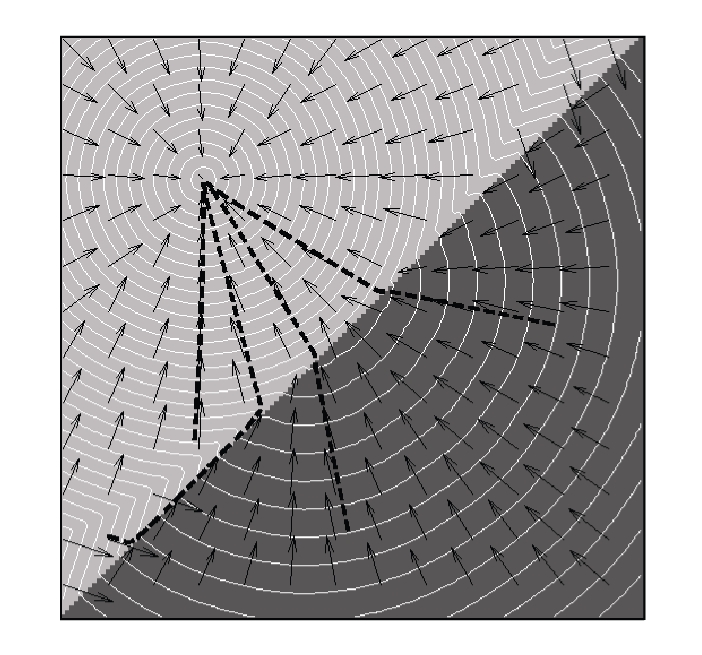
Example geodesics in a double isotropic space. Black arrows show the local orientations of the geodesics. The speed in the dark grey region is twice as high as that in the light grey one. Notice that in each separate space, the geodesics are straight lines. Also, notice how one of the geodesics (bold dashed lines) travels backward to the high speed part before getting back to the low speed one.

Now, let u(x) be the arrival time function starting from a location $x_{0}$, that is, u(x) is equal to the minimum value of the integral J(γ) along a geodesic connecting $x_{0}$ to x. Then, the arrival time function and the geodesics satisfy these two fundamental equations:

(2a) $$\begin{array}{c} \nabla u^{T}M^{-1}\nabla u=1 \end{array},$$

(2b) $$\begin{array}{c} \gamma^{\prime }\propto M^{-1}\nabla u, \end{array}$$

where ∇u is the spatial gradient of u. Equation (2a) is the anisotropic version of the so-called Eikonal equation. In the isotropic case, this equation is usually written |∇u|=1/v, where v is the local speed. Hence, this equation tells us two things: (i) it is a generalisation of the speed equation, stating that the time of arrival is inversely proportional to the speed, and (ii) changing the local metric tensor can be seen as changing the local speed. Equation (2b) shows that the tangent of the geodesic lines is parallel to the gradient of the time of arrival function with respect to the inverse metric. This is very important because it gives us a convenient way to reconstruct geodesics from any point in space, given the solution to the Eikonal equation. Figure 1 shows example geodesics in an isotropic space composed of two subsets with different local speeds.

**[Proof of equation (2)] 2.1**

Recall that the function u(x) is the minimum value of J along the geodesic from point $x_{0}$ to an arbitrary point x:

(3) $$u(x)=\underset{\gamma }{\min}\int_{x_{0}}^{x}F(s,\gamma ,\gamma^{\prime })ds.$$

A general variation of (3) is given (see, e.g., [31]) as

(4) $$\delta u=\frac{\partial F}{\partial \gamma^{\prime }}\delta \gamma +\int_{x_{0}}^{x}\left(\frac{\partial F}{\partial \gamma }-\frac{d}{ds}\frac{\partial F}{\partial \gamma^{\prime }}\right)ds.$$

Since we have integrated along a geodesic, the second term on the right-hand side of (4) equals zero (Euler condition). We obtain

(5) $$\nabla u=\frac{\partial u}{\partial \gamma }=\frac{\partial F}{\partial \gamma^{\prime }}=\frac{M\gamma^{\prime }}{{\left({\gamma^{\prime }}^{T}M\gamma^{\prime }\right)}^{1/2}}.$$

Equation (2b) directly follows. Finally, and using the symmetry of the metric tensor M, we get the Eikonal equation:

(6) $$\nabla u^{T}M^{-1}\nabla u=\frac{{\gamma^{\prime }}^{T}M^{T}M^{-1}M\gamma^{\prime }}{{\gamma^{\prime }}^{T}M\gamma^{\prime }}=1.$$

Equations (2a) and (2b) summarise the two steps for building geodesics: (i) solve the Eikonal equation for u, given a metric tensor M and a starting point $x_{0}$; (ii) construct geodesics between any given point and the starting point $x_{0}$ by following the gradient of u with respect to the inverse metric $M^{-1}$.

### 2.2. Fast-marching algorithm

A few algorithms have been proposed in the literature for computing the function u on a discrete grid. The most popular are Tsitsiklis's method [28] and Sethian's method [32], which are based on the construction of the time of arrival function u(x) using front propagation. These methods are also referred to as fast marching methods because they construct the function u in a single-pass through the grid nodes. Tsitsiklis's method relies on (1) while Sethian's method uses the Eikonal equation (2a). Both methods are suitable in the case of isotropic media, that is, where the metric M is proportional to the identity matrix, but they fail in anisotropic media [23]. An exact scheme to deal with anisotropy has been proposed by Sethian and Vladimirsky [30], but while remaining a single-pass algorithm, it still requires a computational effort that is growing with the amount of anisotropy. A variant of the initial fast-marching algorithm of Tsitsiklis [28] has been proposed to deal with anisotropic media [29], which is more computationally efficient than the exact scheme of Sethian [30]. Yet, it relies on a generic optimisation procedure that was undocumented for the special case of the elliptical media we face with DTI tractography. We extended this method by deriving a solution to the optimisation procedure in this case.

**Figure 2:**
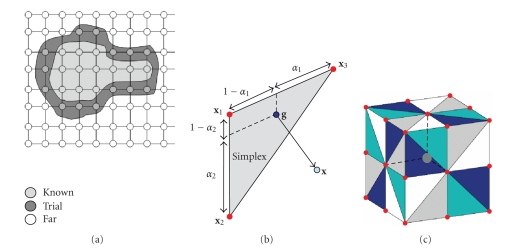
(a) Grid representation of the different sets involved during the fast-marching algorithm. (b) Position of the optimal point on a simplex such as to minimise the geodesic distance to x. (c) Geometry of the 48 simplexes surrounding a voxel (central grey dot). The little red dots represent the centres of the 26 neighbouring voxels.

The general idea of the fast-marching algorithm was borrowed from the graph theory. It is a direct extension of Dijkstra's algorithm for finding minimal paths in a graph [33]. The algorithm relies on a very simple observation: suppose that the time of arrival is known inside a close set of grid nodes (a set we will refer to as the *known* set). Then, the first nodes that will be encountered by the propagating front are the nodes on the *edge* of the *known* set (this narrow band of grid nodes will be called the *trial* set). Secondly, the first node that will be encountered by the propagating front is the closest one to *known* (in terms of geodesic distance), and crucially, there will be no other way to make this distance smaller after propagating the front further. This means that the arrival time at this voxel will not change, and can be *frozen*. In other words, the value of the time of arrival u can be calculated, starting from $x_{0}$, in a single-pass through the voxels, only by considering, at each iteration, the neighbouring voxels of the propagating front. The other voxels (the *far* set) are not examined. Figure 2(a) schematises this front propagation scheme. The fast-marching algorithm is summarised in the appendices.

The crucial step in this front propagation is the computation of the distance between the front and the neighbouring voxels in the *trial* set. In our case, this distance is anisotropic, and we cannot use the standard methods, because they rely on the assumption that the gradients of u are parallel to its geodesic lines (see [23] for further details). To account for the anisotropy, we consider a set of simplexes (triangles) that cover the whole neighbourhood around a voxel of the narrow band [29], and minimise the distance function between the simplexes and that voxel (see Figures 2(b) and 2(c)). The introduction of these simplexes allows to describe the trajectories on a continuous rather than a discrete grid. The definition of a simplex neighbouring a point x is simply a set of three points $\left(x_{1},x_{2},x_{3}\right)$ that are 26 neighbours of x, defining a triangle that we denote $\bar{x_{1}x_{2}x_{3}}$. There are 48 such triangles around x for the 26 connexities (Figure 2(c)). The procedure for computing the anisotropic distance between the propagating front and the voxels in the *trial* set is given in the appendices.

During the updating procedure, the time of arrival at a voxel $x_{m}$ of the *trial* set is calculated from its neighbours on a simplex using an approximation (strictly speaking, two approximations!). Normally, if the geodesic passing by $x_{m}$ comes from simplex $\bar{x_{1}x_{2}x_{3}}$, then the time of arrival is given by

(7) $$u(x_{m})\underset{g\in \overset{¯}{x_{1}x_{2}x_{3}}}{\min}\{u(g)+\int_{g}^{x_{m}}F(s,\gamma ,\gamma^{\prime })ds\}.$$

We use a parametric approximation to this formula, given by the minimisation of the following function:

(8) $$f(\alpha )=\underset{\left(\text{I}\right)}{\underset{︸}{\overset{3}{\underset{i=1}{\sum }}\alpha_{i}u(x_{i})}}+\underset{\left(\text{II}\right)}{\underset{︸}{{\left\|x-\overset{3}{\underset{i=1}{\sum }}\alpha_{i}x_{i}\right\|}_{M}}},$$

where $\|\cdot {\|}_{M}$ is the quadratic norm with respect to the metric M and $\alpha =(\alpha_{1},\alpha_{2},\alpha_{3})$. Equation (8) follows the approximations of Tsitsiklis [28]. Term (I) approximates the distance from the starting point $x_{0}$ to the simplex centre of mass g as a weighted sum of the distances to the nodes of the simplex. Term (II) approximates the remaining distance by considering the local metric as being constant, equal to its value at $x_{m}$.

Minimising f in the simplex can be written as a constrained optimisation problem that can be solved explicitly, since f and the simplex are convex. The analytical solution is detailed in the appendices.

### 2.3. How to choose the metric?

In the GT framework, we make the hypothesis that white matter fibres are geodesics with respect to a metric tensor. But so far, we have not specified which metric tensor we mean. In DTI, the inverse tensor ($M=D^{-1}$) seems to be the natural choice. Intuitively, water molecules diffusion is faster along the tract than across them. When inverting the diffusion tensor, the highest eigenvalues become the lowest, and the shortest distance is parallel to the fibres. One can also notice that the inverse tensor defines a metric in a Riemannian space that induces a Laplace-Beltrami operator (generalisation of the Laplace operator) which is encountered in the diffusion equation [25, 34].

**Figure 3:**
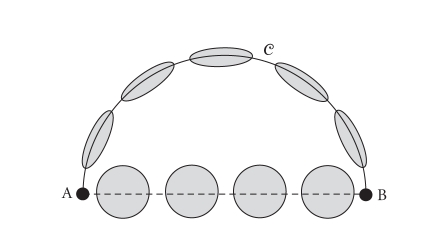
Comparison between a straight line and a geodesic.

**Figure 4:**
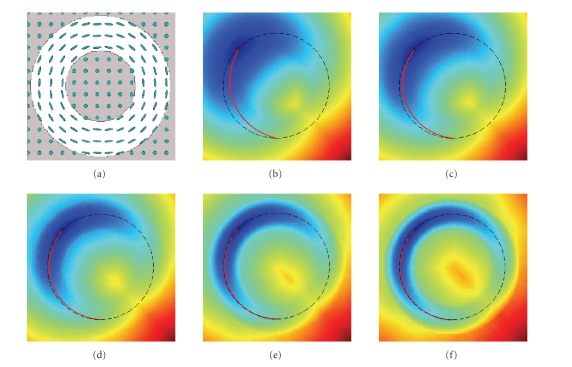
(a) Simulated circular tensor field. (b)–(f) Increasing the anisotropy of the circular tensor makes the geodesic path (red line) closer to a circle.

However, the inverse tensor is not suitable in all circumstances. Consider the situation described in Figure 3 were a circular tract of radius r connects points A and B, with diffusion tensors tangent to the tract having the same shape. Suppose the rest of the space is isotropic, with the same mean diffusion as along the tract. If one considers the inverse tensor metric $M=D^{-1}$, the distance between A and B through the circular path is

(9) $$\int_{C}\sqrt{dx^{T}D^{-1}dx}=\frac{\pi r}{\sqrt{\lambda_{1}}},$$

where $\lambda_{1}$ is the largest eigenvalue of the tensors along the circular pathway. On the other hand, the straight line distance between A and B is equal to $2\sqrt{3}r/\sqrt{\text{trace}(D)}$. Hence, a necessary condition for the circular tract to be a geodesic is that its length is smaller than a straight line, that is,

(10) $$\frac{\pi r}{\sqrt{\lambda_{1}}}\leq \frac{2\sqrt{3}r}{\sqrt{\text{trace}(D)}},$$

which leads to $\lambda_{1}\geq \pi^{2}\text{trace}(D)/12$, that is, a condition on the tensor shape to be peaky enough. Of course, one can imagine that even if this condition is satisfied, a geodesic path might certainly lie somewhere in between a straight line and the circular line, as shown in Figure 4. Which metric to choose is hence still debatable. Nonetheless, in our simulations and real data applications, we will use the inverse diffusion tensor as a metric for defining geodesics.

## 3. APPLICATIONS

### 3.1. Simulations

We have evaluated the GT method on simulated data. The purpose of these simulations is twofold. First, they show how the anisotropic fast-marching algorithm performs on elliptic media, in both homogeneous field (where the analytical solution is available) and a heterogeneous field. Second, they allow to compare GT with streamlining in cases where the data present local perturbations (crossing fibres).

In a homogeneous medium, where the data support the same diffusion tensor D in every voxel, the analytic solution to the Eikonal equation is given by

(11) $$u(x)=\sqrt{\left({x-x_{0})}^{T}D^{-1}(x-x_{0}\right)}.$$

**Figure 5:**
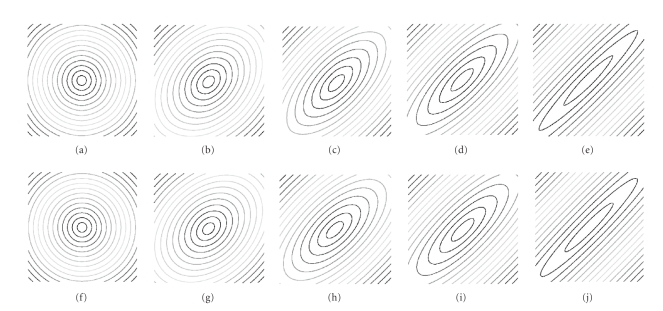
Contour plots of the numerical solution (top) and the analytic solution (bottom) to the Eikonal equation in a homogeneous medium. Anisotropy levels are increasing from left (isotropic) to right (ratio of 50 between the extreme tensor eigenvalues).

It is easy to check that in this case, $u(x_{0})=0$ and $\nabla u^{T}D\nabla u=1$. We generated a tensor where the two smaller eigenvalues are equal, and gradually increased the anisotropy. Figure 5 shows the level curves of the analytic versus the numerical solution to the Eikonal equation. The two solutions are very close even for a large anisotropy, corresponding to a ratio of 50 between the largest and the lowest tensor eigenvalues. Table 1 summarises the mean and standard deviations of the relative error for different values of the anisotropy, which is expressed both in terms of the ratio between the largest and the lowest tensor eigenvalue, or in terms of the more widely used fractional anisotropy (FA, see, e.g., [35]).

In a heterogeneous medium, such an analytical solution does not exist. However, we can verify that the Eikonal equation is satisfied, that is, $\nabla u^{T}D\nabla u$ is equal to one. We used the same circular tensor field as shown in Figure 4. In Table 1, we show the mean and standard deviations of $\nabla u^{T}D\nabla u$ for different anisotropies. Notice that these are close to one, but with a higher deviation from one with increasing anisotropy.

**Figure 6:**
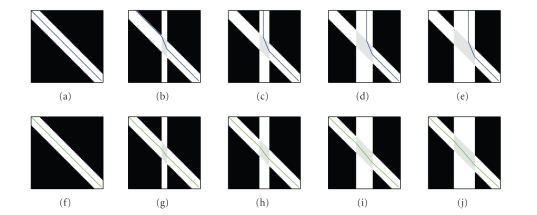
Comparison between streamline (top) and geodesic (bottom) tractography in the presence of a crossing fibre bundle, the width of which increases from zero (left) to twice the width of the principal bundle (right). Note how streamlining gets deviated from the straight line because of partial volume effect.

Finally, we show results of GT in the case of local perturbations. We generated a tensor field simulating a crossing fibre situation. The zone where the two fibres cross has a diffusion tensor that is the average of the two crossing fibres' tensors. We increased the crossing fibre area and compared the behaviour of GT to streamlining tractography (Figure 6). As expected, because the streamlining simply follows the direction of highest diffusion given by the tensor, the fibre trajectory was deviated. In the case of GT, there was little, if any, deviation from the straight line.

### 3.2. Real data

**Acquisition**

Data from a single healthy subject were acquired at *Service de Neuroradiologie (CHNO des Quinze-Vingts, Paris)*. Six gradient weighted and one T_2_-weighted images were acquired on a 1.5 Tesla MR Scanner (GE Signa) using the following scan parameters: 128×128 image matrix, 2.03 mm in-plane pixel size; 3.5 mm slice thickness; b=1000; (TR; TE) = (5000; 91.8)  milliseconds; Number of averages = 8. Thirty-six contiguous slices covering the whole brain were acquired. The total scanning time was approximately 14 minutes.

**Regions of interest**

**Figure 7:**
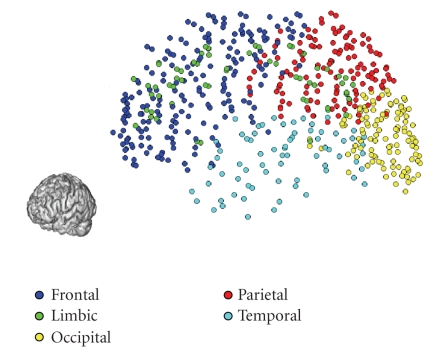
Localisation of the regions of interest on the cortex. 3D fronto-sagittal view.

Five hundred and sixty-seven (N=567) regions covering the whole cortex were manually selected in the DTI space. Each region was represented by a single voxel. The anatomical localization of these regions is shown in Figure 7. We performed a front propagation from each region, which provided the distance functions ${\left(u_{i}\right)}_{i=1}^{N}$. Then back propagation allowed us to construct the N(N−1)/2=160,⁢461 geodesics connecting the whole set of voxel pairs. We computed a heuristic connectivity index consisting of the mean diffusivity along each geodesic, multiplied by the mean FA along the pathways.

**Figure 8:**
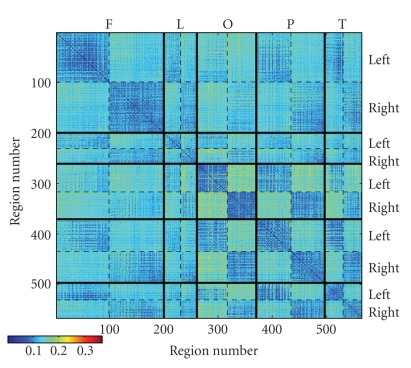
Anatomical connectivity matrix rearranged into anatomical groups: F (frontal lobe), L (limbic), O (Occipital), P (parietal), T (temporal). In each group, the left and right hemispheres are also separated.

In order to better visualize this anatomical connectivity index in a matrix form, the set of brain regions were grouped with respect to their localization. The regions were divided into five groups, including the frontal lobe (left: 99 voxels, right: 101 voxels), the limbic cortex (left: 31, right: 30), the occipital lobe (left: 56; right: 54), the parietal lobe (left: 64; right: 62), and the temporal lobe (left: 34; right: 36). This classification was based on an automatic labelling of the voxels locations given by the Talairach Daemon (http://ric.uthscsa.edu/projects/tdc), after registering the DTI data into the MNI standard space, and subsequent correction from MNI to Talairach space (see, e.g., [36]). Figure 8 shows the distribution of the connectivity index, in the matrix form, between any two regions, arranged by group and by hemisphere.

The matrix shown in Figure 8 reveals an organization of the connectivity index that follows the anatomical organization of the brain regions regarding their locations. Since the connectivity index encompasses the anisotropy factor, its value highly depends on which regions we are connecting, which means which global pathways the geodesics are close to.

First, the diagonal blocks of the matrix show clearly a lower level of connectivity than the extradiagonal blocks. This seems to indicate that the connectivity index penalises short fibers, and inversely favors long fibers, especially interhemispheric fibers. Secondly, the blocks that show the highest connectivity index are the blocks that connect the right and left occipital lobes.

This result is not surprising since the fiber tracts that connect right and left occipital lobes follow a trajectory through the splenium of the corpus callosum (forceps major), which is a highly anisotropic area.

**Geodesics**

**Figure 9:**
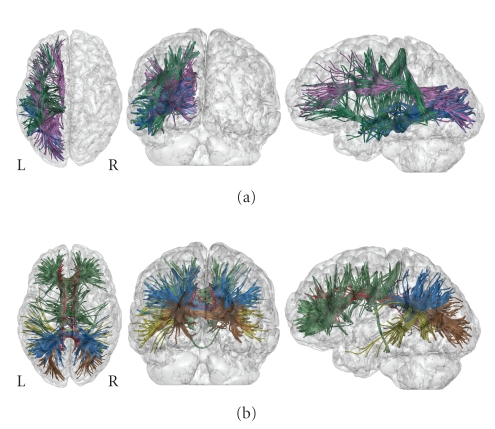
(a) 10% most probable intrahemispheric geodesics shown in the left hemisphere. Blue paths connect the occipital lobe to the temporal lobe. Purple paths connect the frontal to the occipital lobe. Green paths connect the frontal lobe to the temporal lobe. (b) 10% most probable interhemispheric geodesics connecting symmetrical regions. Green: frontal lobe, red: limbic lobe, brown: occipital lobe, blue: parietal lobe, yellow: temporal lobe.

We further investigated which of the constructed geodesics may represent actual fiber trajectories. To approach this question, we thresholded the connectivity matrix in order to emphasize the geodesics with the largest connectivity indices. Specifically, we considered the 10% geodesics with the highest connectivity indices for each interhemispheric block connecting symmetrical groups, taken independently. Figure 9 represents each group of geodesics in different colors. The most probable geodesics paths follow the principal long association fasciculi. The frontal lobe is connected to the occipital lobe via the fronto-occipital fasciculus. The temporal lobe is connected to the occipital via the inferior longitudinal fasciculus, and to the frontal lobe via the uncinate fasciculus. All major long association tracts are represented by these geodesics.

**Geodesics versus streamlining**

**Figure 10:**
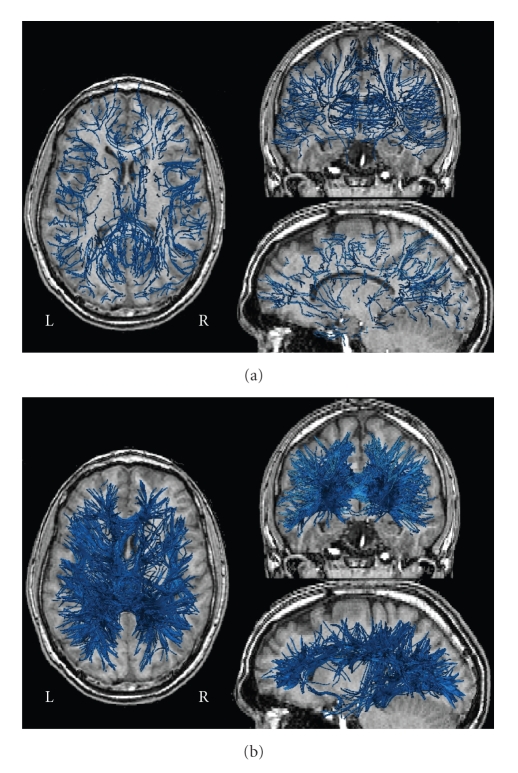
(a) Results of the streamline tractography algorithm applied to the set of brain voxels. Four streams per voxel are computed. The stopping criteria are $60^{\circ }$ for the maximal angle step, and 0.1 for the minimal anisotropy value. (b) Geodesics computed by the GT method. For each brain voxel of the set, four geodesics with the highest probability index are shown.

Finally, in order to compare the results of our method to a conventional fiber tracking method, we performed a streamline tractography from the N seed voxels, with four tracts per voxel. As a stopping criterion, we chose a maximum step angle of 60° and an anisotropy threshold of 0.1 [19]. To compare the results to GT, we selected the four geodesics, having the highest probability index, for each voxel in the set of seed voxels. This way, we have the same number of tracts using both methods (4×N tracts). Figure 10 shows the results of these two procedures. The streamline method produces many incomplete tracts, especially association tracts, while the proposed GT method succeeded in reconstructing the major association and commissural tracts, including the uncinate, the inferior fronto-temporal, and the callosal fibers. Note that the fronto-occipital tract is not present at this level of threshold (we only considered four geodesics per voxel).

## 4. DISCUSSION

Global optimisation is a valuable strategy in the context of path planning. When one has the information of where to start and where to go, this information is used to overcome local poor optimality. In the context of white matter diffusion-based tractography, where we often have strong hypotheses about the localisation of the regions in the brain, global optimisation can overcome some serious weaknesses of the process. Mainly, uncertainty about local fibre orientation, reflecting partial volume effects caused by crossing fibres, or local low signal to noise, can be handled efficiently using GT.

We have presented here a method to perform such global-based path planning in an anisotropic medium. The method is very robust to high anisotropy, and provides an extremely accurate numerical solution to the Eikonal equation.

On real-data experiments, the reconstructed geodesics that have a high connectivity index correspond to known fiber tract fasciculi connecting the cortex. These fasciculi can all be retrieved by other tractography methods that use DTI data, providing priors on their location using one or more regions of interest [37, 38], especially intermediate regions located in white matter. GT automatically depicted these fasciculi with no prior.

However, the U-shaped fibers, that is, the short association tracts, are not favored by our connectivity index. This can be easily seen by looking at the diagonal blocks of the matrix in Figure 8. The long association tracts, as well as the commissural fibers, are more present with a higher connectivity index.

GT also allows one to construct interhemispheric tracts between each pair of regions located in different hemispheres. These tracts include homotopic and heterotopic connexions, that is, tracts connecting, respectively, symmetrical and asymmetrical regions lying in different hemispheres. It is worth noting that standard tractography methods usually fail to recover most callosal connexions, apart from the medial ones. This is a good illustration of the problem of crossing fibres, as those connexions cross the superior longitudinal fasciculus. However, recent probabilistic tractography with more complex local models has successfully traced those types of connexions [14, 20, 21].

There is an intuitive relationship between geodesic, for the inverse tensor metric, and probabilistic tractographies. Probabilistic tractography consists of constructing a distribution of connexions, by sampling tracts using local orientation distributions. In the basic case where this local probability model for fibre orientations is defined using the tensor model (i.e., a Gaussian local model with a covariance matrix proportional to the diffusion tensor D), the probability of a tract following an orientation given by dx at a location x writes

(12) p(x+dx∣x)=N(x,D),

then, for some pathway γ connecting $x_{0}$ to $x_{1}$, and for some discretisation of this pathway, the probability of moving along γ is the product of the infinitesimal step probabilities:

(13) $$\begin{array}{cc} p{(x}_{0}⟶x_{1}) & =\overset{n}{\underset{k=1}{\prod }}p(x_{0}+kdx|x_{0}+(k-1)dx)\\ & \propto \overset{n}{\underset{k=1}{\prod }}\exp\left\{-\frac{1}{2}dx^{T}D^{-1}dx\right\}\\ & =\exp\left\{-\frac{1}{2}\overset{n}{\underset{k=1}{\sum }}dx^{T}D^{-1}dx\right\}\\ & ⟶\exp\left\{-\frac{1}{2}\int_{\gamma }dx^{T}D^{-1}dx\right\}\\ & \leq \exp\left\{-\frac{1}{2}{\left(\int_{\gamma }\sqrt{dx^{T}D^{-1}dx}\right)}^{2}\right\}. \end{array}$$

Maximising this probability could then be related to minimising the geodesic distance, relative to the inverse tensor metric. While the probabilistic method gives a distribution of connexions, GT gives the mode of this distribution, that is, the path with highest probability. Note also that the probabilistic model given by (12) can be improved to fit the data more accurately (e.g., multiple tensors, etc.), which can be seen as a change in the metric tensor in GT.

Using GT, it is possible to study the organisation of large brain networks in terms of their anatomical connexions. Such networks have been studied in terms of structural invariants in a graph theoretical framework by several authors [39–41]. These works have been conducted for studying the structural organisation of the cat or primate brain, as well as for the human functional brain organisation, but have never been applied to large human anatomical networks, because no method has been proposed to construct such networks. GT could provide this structural information, via a graph that has been thresholded or not, since the connectivity index in itself contains information about the connectional structure.

There are two major issues when using geodesics for the tractography. First, choosing a metric for which geodesics represent fibre pathway trajectories is not straightforward. The correct metric might show more anisotropy than the diffusion tensor, as discussed earlier. Also, the choice of the metric might depend on the white matter fibres under investigation. The second issue is that, for any pair of regions in the brain, there exists a geodesic between those regions. However, this is not true for white matter fibres. One then has to decide when a geodesic is a fibre trajectory, for example, by defining indices and performing statistical thresholding under some null hypothesis. This problem of thresholding tractography results is not specific to GT, but is met by any other tractography method. It is though a bigger problem in the case of GT because every pair of regions is potentially connected. Another problem with GT is that, in the presence of two separate connexions between two regions, we are only able to detect one of them (the shortest one in terms of geodesic distance).

One way to validate GT results would be by comparison with another measure of connectivity. For example, measures of functional connectivity using functional magnetic resonance imaging (fMRI) by means of correlations [42] or partial correlations [43] are thought to be closely linked to the anatomical structure sustaining the brain regions, seen as graph nodes. The GT technique provides a unique tool for performing a comparison between anatomical and functional connectivity, since it can apply to large networks, and provide a measure of anatomical connectivity between each pair of nodes of the brain network. It can readily be used to compare the architectures of brain networks that have been studied in humans from the functional perspective (e.g., Salvador et al. [44] used partial correlations of fMRI data on a set of 100 regions), or using voxel-based morphometry to correlate cortical thickness between different cortical areas (e.g., He et al. [45] used this technique to study 100 cortical areas in humans). Such investigations have considerable possible applications, both cognitive and clinical. On the one hand, this method could serve as a basis for comparing anatomical and functional connectivities, as said earlier, and could help to understand how the brain works as an evolving network. On the other hand, the structure of restricted networks has already helped to distinguish between healthy subjects and patients, for example, Alzheimer disease in the case of functional connectivity [46], and Schizophrenia in the case of white matter morphology [47]. The GT method could serve for the characterisation of the structural organisation of those brain networks in terms of their connectional fingerprints.

## Figures and Tables

**Table 1:** Summary of the simulation results with an increasing ratio between the largest and the lowest tensor eigenvalue (the corresponding FA value is shown on the second row). Top: mean and standard deviations of the relative error between numerical and analytic solutions for the Eikonal equation in a homogeneous medium. Bottom: mean and standard deviations of the value of $\nabla u^{T}D\nabla u$ in a circular tensor field.

ratio	1	2	5	10	50
FA	0	0.17	0.59	0.79	0.96
mean (%)	0.79	0.93	1.25	1.54	2.16
SD (%)	0.62	0.86	1.53	2.16	3.71

ratio	5	10	20	50	100
FA	0.59	0.79	0.90	0.96	0.98

mean	0.995	0.993	0.989	0.997	1.059
SD	0.068	0.086	0.112	0.213	0.634

## APPENDICES

### A. ALGORITHMS

Algorithm 1 Fast marching algorithm

Definitions 1 Let *Known* be the set of points whose u-value has been computed and will not change. Let *Trial* be the set of voxels that are being examined (26-neighbourhood of *Known*), and let *Far* be the set of voxels that have not been examined yet. Finally, if S is a set of voxels, let #S denote the number of voxels that belong to S.

- Initialization:
  - move $x_{0}$ to *Known* and set $u(x_{0})=0$,
  - move to *Far* every x such that $x\neq x_{0}$ and set u(x)=∞,
  - update u in the neighbourhood of $x_{0}$ using Algorithm [Statement alg2],
- While #⁢Trial ≠ 0:
  - search for the voxel $x_{m}$ in *Trial* with the smallest value of u,
  - move $x_{m}$ to *Known*,
  - update u in the neighbourhood of $x_{m}$ using Algorithm [Statement alg2].

Algorithm 2 Updating procedure for the distance function u at voxel $x_{m}$:

- for all x in the 26-neighborhood of $x_{m}$ and x∉Known,
  - if x∈Far, move x to *Trial*,
  - for all $\bar{x_{1}x_{2}x_{3}}$ surrounding x,
    1. compute $u^{*}(x)=\min_{0\leq \alpha_{i}\leq 1;\sum \alpha_{i}=1}f(\alpha )$,
    2. $u(x):=\min\{u^{*}(x),u(x)\}$.

### B. EXPLICIT SOLUTION FOR THE UPDATING PROCEDURE

Here we provide an explicit solution for the minimisation problem formulated in (8). Recall that the problem was to find the minimum, inside a simplex, for the following expression:

B.1 $$\begin{array}{ll} \underset{\alpha }{\min}\,\;f(\alpha ) & =\overset{3}{\underset{i=1}{\sum }}\alpha_{i}u(x_{i})+\|x-\overset{3}{\underset{i=1}{\sum }}\alpha_{i}x_{i}{\|}_{M}\\ \alpha \in \Delta & =\{(\alpha_{1},\;\alpha_{2},\;\alpha_{3})\in [0,1{]}^{3}/\alpha_{1}\,+\;\alpha_{2}+\;\alpha_{3}=1\}. \end{array}$$

In order to simplify the notations, and considering that $\alpha_{3}=1-\;\alpha_{1}-\alpha_{2}$, we will use the following:

B.2 $$\begin{array}{ll} k_{1:2} & =u(x_{1:2})-u(x_{3}),\;\;k_{3}=u(x_{3}),\\ y_{1:2} & =x_{3}-x_{1:2},\;\;y_{3}=x_{3}-x,\\ r_{ij} & =y_{i}^{T}My_{j}. \end{array}$$

The function u depends simply on $\alpha_{1}$ and $\alpha_{2}$:

B.3 $$f(\alpha )=\varphi (\alpha_{1},\alpha_{2})=\alpha_{1}k_{1}+\alpha_{2}k_{2}+k_{3}+{\left\|\alpha_{1}y_{1}+\alpha_{2}y_{2}+y_{3}\right\|}_{M}.$$

φ is differentiable and convex, it is then minimal when Δφ=0. When constraining the minimum to lie inside the simplex Δ, the solution is either that for which the gradient is zero, if it lies inside Δ, or it is on the edges of Δ if the gradient is zero outside the simplex. In the latter case, the minimisation problem is 1D, and the solution simplifies greatly.

First, let us write the unconstrained solution: ∇φ=0 implies

B.4 $$\begin{array}{cc} \underset{A_{1}}{\begin{array}{c} \underset{︸}{\begin{array}{c} \left(k_{1}r_{12}-k_{2}r_{11}\right) \end{array}} \end{array}} & \cdot \alpha_{1}+\underset{A_{2}}{\underset{︸}{\left(k_{1}r_{22}-k_{2}r_{12}\right)}}\\ & \cdot \alpha_{2}+\underset{B}{\underset{︸}{\left(k_{1}r_{23}-k_{1}r_{13}\right)}}=0. \end{array}$$

This equation means that the minimum of φ lies on the straight line defined by the equation $A_{1}x+A_{2}y+B=0$. This simplifies the problem, as the problem is again 1D if we replace $\varphi (\alpha_{1},\alpha_{2})$ by the function $\overset{˜}{f}(\alpha )$ which expression depends on the values of $A_{i}$ and B.

- If $A_{1}=A_{2}=0$,
  B.5 $$\begin{array}{cc} \alpha_{1} & =\frac{r_{12}r_{23}-r_{13}r_{22}}{r_{11}r_{22}-r_{12}^{2}},\\ \alpha_{2} & =\frac{r_{13}r_{12}-r_{23}r_{11}}{r_{11}r_{22}-r_{12}^{2}}. \end{array}$$
- If $A_{j}\neq 0$,
  B.6 $$\begin{array}{cc} \alpha_{i} & =\underset{\alpha }{\text{argmin}}\{\alpha (k_{i}-\frac{A_{i}}{A_{j}}k_{j})-k_{j}\frac{B}{A_{j}}+u_{3}\\ & \;\;\;\;\;+{\left\|\alpha x_{i}-\frac{A_{i}}{A_{j}}x_{j}-\frac{B}{A_{j}}x_{j}+x_{3}\right\|}_{M}\}\\ \alpha_{j} & =-\frac{A_{i}}{A_{j}}\alpha_{i}-\frac{B}{A_{j}}. \end{array}$$

In the last case, the problem reduces to minimising a 1D function of the form $\overset{˜}{f}(\alpha )=\alpha k+u+\|{\alpha z_{1}+z_{2}\|}_{M}$, in which case the solution writes

B.7 $$\alpha =-\frac{\left(r_{12}+k\sqrt{\left|R|/(r_{11}-k^{2}\right)}\right)}{r_{11}},$$

where $r_{ij}=z_{i}^{T}Mz_{j}$ and $|R|=r_{11}r_{22}-r_{11}^{2}$.

Finally, if the solution given by the above lies outside the simplex (i.e., $|\alpha_{i}|>0$), then we minimise φ on the edges of the simplex, which is again a 1D problem. This is equivalent to setting one of the $\{\alpha_{i}{\}}_{i=1}^{3}$ to zero, and keeping the results which minimises φ:

- $\alpha_{1}=0$:
  B.8 $$\begin{array}{c} \alpha_{2}=\underset{\alpha }{\text{argmin}}\{\alpha k_{2}+k_{3}+\|{\alpha y_{2}+y_{3}\|}_{M}\}, \end{array}$$
- $\alpha_{2}=0$:
  B.9 $$\begin{array}{c} \alpha_{1}=\underset{\alpha }{\text{argmin}}\{\alpha k_{1}+k_{3}+\|{\alpha y_{1}+z_{3}\|}_{M}\}, \end{array}$$
- $\alpha_{1}+\alpha_{2}=1$:
  B.10 $$\begin{array}{cc} \alpha_{1} & =\underset{\alpha }{\text{argmin}}\{\alpha (k_{1}-k_{2})+k_{2}+k_{3}\\ & \;\;\;\;\;+\|{\alpha (y_{1}-y_{2})+y_{2}+y_{3}\|}_{M}\}\\ \alpha_{2} & =1-\alpha_{1}. \end{array}$$
